# Assessment of Cardiovascular Parameters in Obese Children and Adolescents with Non-Alcoholic Fatty Liver Disease

**DOI:** 10.4274/jcrpe.1949

**Published:** 2015-08-31

**Authors:** Beray Selver Eklioğlu, Mehmet Emre Atabek, Nesibe Akyürek, Hayrullah Alp

**Affiliations:** 1 Necmettin Erbakan University Faculty of Medicine, Division of Pediatric Endocrinology and Diabetes, Konya, Turkey; 2 Konya Training and Research Hospital, Clinic of Pediatric Endocrinology and Diabetes, Konya, Turkey; 3 Malatya State Hospital, Clinic of Pediatric Cardiology, Malatya, Turkey

**Keywords:** obesity, Non-alcoholic fatty liver disease, periaortic fat thickness, children, adolescent

## Abstract

**Objective:**

The aim of this study was to evaluate the periaortic fat thickness (PAFT) using conventional echocardiography in obese children and adolescents with non-alcoholic fatty liver disease (NAFLD).

**Methods:**

Two hundred and ninety-seven obese children and adolescents were included in the study. Anthropometric measurements were made in all subjects, and fasting venous blood samples were taken for determination of glucose, insulin, total cholesterol, low-density lipoprotein (LDL) cholesterol, high-density lipoprotein (HDL) cholesterol, triglycerides, alanine aminotransferase (ALT) and aspartate aminotransferase (AST) levels. Ultrasonography of the liver was used for assessment of NAFLD and the subjects were grouped as NAFLD and non-NAFLD. Echocardiography was performed in all subjects.

**Results:**

PAFT was higher in patients with NAFLD compared with the non-NAFLD group. In patients with NAFLD, PAFT was positively correlated with waist circumference and with total cholesterol levels. In multiple regression analysis, waist circumference (β=0.28, p=<0.001) was found to be the best predictor of PAFT.

**Conclusion:**

Conventional echocardiography may be used to determine increased PAFT at an early stage in obese children and adolescents with NAFLD for careful monitoring of cardiovascular risk.

## INTRODUCTION

Non-alcoholic fatty liver disease (NAFLD) refers to a spectrum of hepatic pathologies that resemble alcoholic liver disease but without alcohol consumption. Its clinico-pathological spectrum ranges from simple steatosis to steatohepatitis, fibrosis and cirrhosis of the liver (1). NAFLD is often associated with obesity, type 2 diabetes mellitus, dyslipidemia and hypertension, i.e. abnormalities which carry a cardiovascular disease risk. NAFLD is accepted to be a hepatic manifestation of obesity. Studies report the prevalence of NAFLD to be up to 15-30% of the general population (2,3).

NAFLD patients have a higher prevalence of atherosclerosis, as shown by increased carotid-wall intimal thickness, increased numbers of atherosclerotic plaques and increased plasma markers of endothelial dysfunction. Pathologic studies have shown that atherosclerosis is an early process beginning in childhood, with fatty streaks observed in the aorta and the coronary and carotid arteries in children and adolescents (4).

One component of abnormal body fat deposition involves the deposition of adipose tissue, so-called ectopic fat, around organs and the vasculature. Perivascular fat is one such ectopic fat depot that has been postulated to have a local pathogenic effect on blood vessels. Periaortic fat is a subtype of perivascular fat and can now be quantified using multidetector computed tomography, but only in adults (5,6).

An association between NAFLD and atherosclerotic risk factors is generally observed in adults and there are a few studies concerning carotid intima-media thickness and NAFLD (7). However, there is no study addressing periaortic fat thickness (PAFT) and NAFLD.

We aimed to investigate the association between PAFT and NAFLD in obese children and adolescents. To our knowledge, the present study is the first to show this link.

## METHODS

Two hundred and ninety-seven (150 girls and 147 boys, aged 11.8±2.8 years) outpatient children and adolescents were included in the study. All subjects had body mass index (BMI) values greater than the 95th percentile for age and gender based on the Turkish children’s percentile curves (8). Patients who had major illness such as type 1 or 2 diabetes, a history of liver disease and those who were on medications were excluded. The patients were divided into two groups as those with NAFLD and those without NAFLD; the parameters examined were compared between these two groups. The study was approved by the local ethics committee (2013/485) and was conducted in accordance with the guidelines proposed in the Declaration of Helsinki.

A comprehensive physical examination including anthropometric measurements was made in all subjects. Puberty was assessed by using the criteria of Tanner stages (9,10). Tanner stages 2, 3, 4, 5 were considered as pubertal. Waist circumference (WC) was measured at the level of the umbilicus. WC was evaluated using the percentile curves for WC of healthy Turkish children (11). Blood pressure was measured. Blood pressure threshold values were used in reference to the National High Blood Pressure Education Program Working Group in 2004 with the normal values reported for children (12).

Serum fasting glucose, fasting plasma insulin, alanine aminotransferase (ALT), aspartate aminotransferase (AST), total cholesterol, triglyceride (TG), low-density lipoprotein (LDL), and high-density lipoprotein (HDL) cholesterol levels were determined. An ALT value >55 IU/L was considered elevated.

Dyslipidemia was defined as a HDL level of <40 mg/dL, a total cholesterol level ≥200 mg/dL, a LDL level ≥130 mg/ dL, and a TG level of ≥100 mg/dL for ages 0-9 and ≥130 mg/dL for ages 10-19 years (13).

The homeostasis model assessment of insulin resistance (HOMA-IR; fasting insulin x fasting glucose/22.5) was used as an index of insulin resistance (14). Insulin resistance is defined as a HOMA-IR of greater than 2.5 in the prepubertal subjects and 3.16 in the pubertal ones (15).

Liver ultrasonography results were used in the assessment of NAFLD.

Echocardiography of the aorta was performed. Measurement of perivascular adipose tissue was made with conventional methods from the adventitia layer of the abdominal aorta, the adventitial layer of the aorta adjacent to the form of the measurement of the linear echogenic line. Both the axial and sagittal planes in the supine position, the L1-2 level (just above the umbilicus), proximal to the iliac bifurcation, measurements were taken as a graph.

### Statistical Analysis

The normality of the data was checked and the data were expressed as mean ± SD. Differences were analyzed using the student’s t-test and the chi-square test. Multiple regression analysis was performed. PAFT measurement reliability test in the obese and control groups were made. Axial and sagittal measurement compliance (reliability) within observers and between observers was examined. Measurements of periaortic thickness were consistent for both evaluators as well as evaluators of the intraclass determined by correlation coefficient (intraclass correlation coefficient-ICC) with 95% confidence intervals. Statistical significance was taken as p<0.05.

## RESULTS

The prevalence of NAFLD was 20.5% in the obese study population. The prevalence of hypertension was 39.1%. The mean age in all study groups was 11.8±2.8 years, and 69% of the children were pubertal. Mean PAFT was 0.260±0.031. Clinical and laboratory characteristics of all patients are shown in Table 1. PAFT was not significantly different according to sex and pubertal stage (p=0.76, p=0.053, respectively). Traditional risk factors such as systolic blood pressure (SBP), diastolic blood pressure (DBP), WC, LDL, total cholesterol, and TG were not statistically different according to sex.

**Figure 1:**
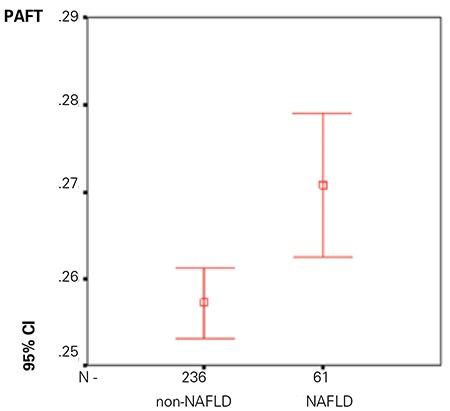
The periaortic fat thickness according to non-alcoholic fatty liver disease

The percentage of girls and boys with NAFLD was 50.2% and 40.8%, respectively. The mean age of the subjects in the NAFLD group was 13.0±2.2 years and 86.8% of them were pubertal. The prevalence of hypertension was 39.3% in the NAFLD group. Serum AST and ALT levels were significantly different between the NAFLD and non-NAFLD patients (p=0.007, p<0.001, respectively). WC and PAFT values were also significantly different between the two study groups (p=<0.001, p=0.003, respectively). The relationship between PAFT and NAFLD is shown in Figure 1. There was no significant difference between sexes for NAFLD. We found a statistically significant difference between groups with and without NAFLD with respect to puberty.

**Figure 2:**
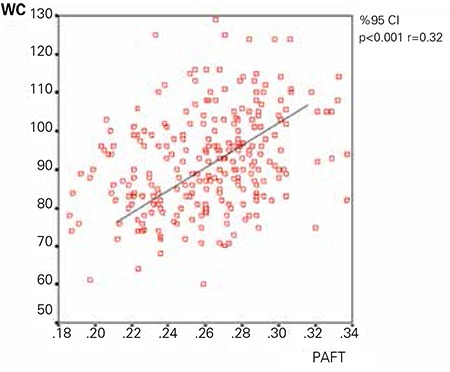
The correlation between periaortic fat thickness and waist circumference

Significant correlations of PAFT are shown in Table 2. The correlation between PAFT and WC was shown in Figure 2.

PAFT significantly correlated with WC and total cholesterol in the NAFLD group (r=0.29, p=0.024; r=0.28, p=0.03, respectively).

In multivariate regression analysis, the only predictor of PAFT was WC (β=0.28, p=0.001) in the NAFLD group.

## DISCUSSION

In this study, we found that NAFLD was associated with increased PAFT in obese children and adolescents. Another important finding of this study was that of all other clinical and laboratory parameters, such as BMI, blood pressure, serum glucose, insulin, and lipids, WC was the only parameter predicting PAFT in the NAFLD group.

Atherosclerosis is thought to begin in childhood and develop silently for decades before clinical events occur (16). The mechanisms by which obesity might contribute to vascular disease remain incompletely understood (5). There is an association between cardiovascular risk factors such as obesity, dyslipidemia, hypertension and early atherosclerotic lesions in the aorta, coronary and carotid arteries (17). Body fat distribution may be a cardiovascular risk factor, even after accounting for generalized adiposity. One component of abnormal body fat deposition involves the deposition of adipose tissue, so-called ectopic fat, around organs and the vasculature. Perivascular fat is one such ectopic fat depot that has been postulated to have a local pathogenic effect on blood vessels. Thoracic periaortic fat is a subtype of perivascular fat (16).

The relationship between NAFLD and atherosclerosis development has been evaluated in pediatric studies (18). NAFLD patients are at an increased risk of developing cardiovascular disease (CVD) since this condition is associated with a number of CVD risk factors including insulin resistance, metabolic syndrome, hypertension, dyslipidemia, type 2 diabetes, and abdominal obesity. In addition, patients with NAFLD have a two-fold risk of CVD mortality, independent of the metabolic syndrome and other established CVD risk factors. Patients with NAFLD have been reported to have an increased risk for CVD as assessed by a variety of outcomes, such as coronary artery calcium scoring, coronary angiography, endothelium-dependent ﬂow-mediated dilation, left ventricular mass index, and c-IMT (19). To our knowledge, there have been no studies showing an association between PAFT and NAFLD in children. Previous adult studies have indicated a consistent association between NAFLD and measures of atherosclerosis and CVD, independent of anthropometric and other CVD risk factors (2,20). In our study, obese children and adolescents with NAFLD had increased PAFT and so might have a greater adverse cardiovascular risk profile than those without NAFLD. This suggested to us that the development and progression of both atherosclerosis and NAFLD may share a similar pathogenesis. Supporting this hypothesis, Loria et al (21) demonstrated that similarities existed between the liver and blood vessels which showed a potential reciprocal inﬂuence in these two organs, leading to the notion of a ‘liver-vessel axis’.

NAFLD includes a spectrum of diseases, from asymptomatic steatohepatitis to cirrhosis. The pathogenesis of NAFLD has remained poorly understood (22). Today, NAFLD is accepted to be a hepatic manifestation of obesity (18). An increased prevalence of NAFLD has been observed along with a dramatic rise in obesity in children during the past three decades. The risk factors for NAFLD are obesity, hyperlipidemia, insulin resistance, and diabetes (18,23). The exact prevalence of NAFLD is unclear. The prevalence of NAFLD is increased to 10-80% in adults with obesity (2). Akın et al (7) found the prevalence of NAFLD in obese children and adolescents to be 35.7%. In our study, the prevalence of NAFLD was 20.3 % in obese patients. Also in our study, the NAFLD group showed a male predominance. In previous studies (24,25), similar results were obtained both in adults and adolescents. These findings show that boys might have a greater tendency to fatty liver.

WC is used for predicting abdominal obesity. In many adult studies, abdominal obesity has been shown to be a particularly important cardiovascular risk factor and it has also been gaining attention in pediatric studies (26). In adult studies, both Fallo et al (27) and Hurjui et al (28) defined WC as a predictor of NAFLD. In agreement with the literature, WC was found to be higher in the NAFLD group also in our study and was positively correlated with PAFT.

TG has long been considered to be a major factor in the development of NAFLD, but there is evidence that non-TG lipid molecules are implicated in the pathogenesis of NAFLD in the process of lipotoxicity. Conversely, the formation of TG may actually be a cytoprotective mechanism in the liver (29).

Similar to the results of previous studies, we did not find significance in the lipid profile of the NAFLD group (29).

The overall incidence of hypertension was higher in our NAFLD groups. We also found insulin levels and BMI to be higher in the NAFLD group. The mechanism might therefore involve elevated insulin levels. Elevated insulin levels in obese patients are believed to stimulate myocyte growth and interstitial fibrosis with sodium retention and activation of the sympathetic nervous system (30). It is possible that the difference between the findings of our study and those of others also relate to differences in the age range and sex of the patients and also to the metabolic control of the study population.

One of the limitations of our study was that the subjects did not represent the general population, but was limited to those who attended our center. Another limitation was that we used abdominal ultrasonography for diagnosing NAFLD although the gold standard is liver biopsy. Although sensitivity is reduced when hepatic infiltration is under 33% (31), some studies do support ultrasonography for NAFLD screening (2).

Our study showed the existence of several cardiometabolic risk factors in obese patients with NAFLD. According to our study, PAFT in NAFLD may be a good predictor of early atherosclerosis, independent of other traditional risk factors. To provide early detection and management of risk factors for CVD complications, PAFT should be considered in children and adolescents with NAFLD.

## Figures and Tables

**Table 1:**
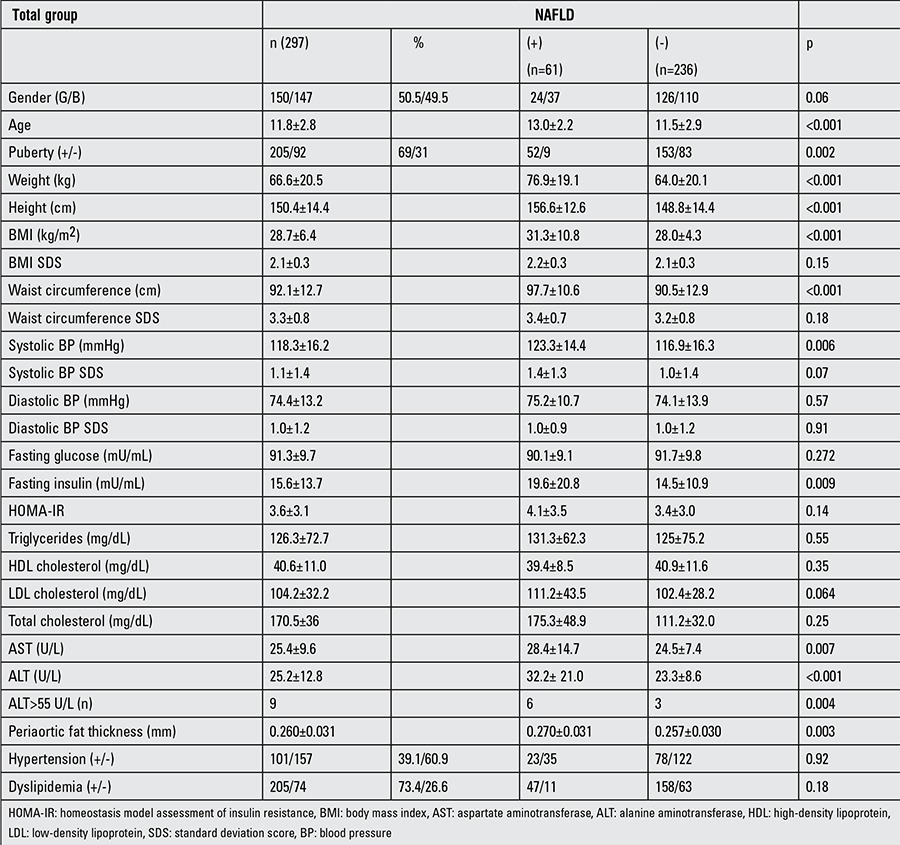
Clinical and laboratory features in the total group and in non-alcoholic fatty liver disease (+) and non-alcoholic fatty liver disease (-) groups

**Table 2:**
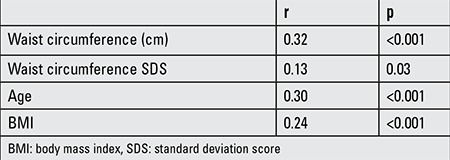
Parameters showing significant correlations with periaortic fat thickness

## References

[1] Fu JF, Shi HB, Liu LR, Jiang P, Liang L, Wang CL, Liu XY (2011). Non-alcoholic fatty liver disease: An early mediator predicting metabolic syndrome in obese children?. World J Gastroenterol.

[2] Hamaguchi M, Takeda N, Kojima T, Ohbora A, Kato T, Sarui H, Fukui M, Nagata C, Takeda J (2012). Identification of individuals with non-alcoholic fatty liver disease by the diagnostic criteria for the metabolic syndrome. World J Gastroenterol.

[3] Donati G, Stagni B, Piscaglia F, Venturoli N, Morselli-Labate AM, Rasciti L, Bolondi L (2004). Increased prevalence of fatty liver in arterial hypertensive patients with normal liver enzymes: role of insulin resistance. Gut.

[4] Pacifico L, Nobili V, Anania C, Verdecchia P, Chiesa C (2011). Pediatric non alcoholic fatty liver disease, metabolic syndrome and cardiovascular risk. World J Gastroenterol.

[5] Britton KA, Pedley A, Massaro JM, Corsini EM, Murabito JM, Hoffmann U, Fox CS (2012). Prevalence, distribution, and risk factor correlates of high thoracic periaortic fat in the Framingham Heart Study. J Am Heart Assoc.

[6] Spiroglou SG, Kostopoulos CG, Varakis JN, Papadaki HH (2010). Adipokines in periaortic and epicardial adipose tissue: differential expression and relation to atherosclerosis. J Atheroscler Thromb.

[7] Akın L, Kurtoglu S, Yikilmaz A, Kendirci M, Elmalı F, Mazicioglu M (2013). Fatty liver is a good indicator of subclinical atherosclerosis risk in obese children and adolescents regardless of liver enzyme elevation. Acta Paediatr.

[8] Bundak R, Furman A, Gunoz H, Darendeliler F, Bas F, Neyzi O (2006). Body mass index references for Turkish children. Acta Paediatr.

[9] Marshall WA, Tanner JM (1970). Variations in the pattern of pubertal changes in boys. Arch Dis Child.

[10] Marshall WA, Tanner JM (1969). Variations in pattern of pubertal changes in girls. Arch Dis Child.

[11] Hatipoglu N, Ozturk A, Mazicioglu MM, Kurtoglu S, Seyhan S, Lokoglu F (2008). Waist circumference percentiles for 7- to 17-year-old Turkish children and adolescents. Eur J Pediatr.

[12] National High Blood Pressure Education Program Working Group on High Blood Pressure in Children and Adolescents (2004). The fourth report on the diagnosis, evaluation, and treatment of high blood pressure in children and adolescents. Pediatrics.

[13] Güngör NK (2014). Overweight and obesity in children and adolescents. J Clin Res Pediatr Endocrinol.

[14] Conwell LS, Trost SG, Brown WJ, Batch JA (2004). Indexes of insulin resistance and secretion in obese children and adolescents: a validation study. Diabetes Care.

[15] Keskin M, Kurtoglu S, Kendirci M, Atabek ME, Yazici C (2005). Homeostasis model assessment is more reliable than the fasting glucose/insulin ratio and quantitative insulin sensitivity check index for assessing insulin resistance among obese children and adolescents. Pediatrics.

[16] Gilardini L, Pasqualinotto L, Matteo S, Caffetto K, Croci M, Girola A, Invitti C (2011). Factors associated with early atherosclerosis and arterial calcifications in young subjects with a benign phenotype of obesity. Obesity (Silver Spring).

[17] Berenson GS, Srinivasan SR, Bao W, Newman WP, Tracy RE, Wattigney WA (1998). Association between multiple cardiovascular risk factors and atherosclerosis in children and young adults. The Bogalusa Heart Study. N Engl J Med.

[18] Pacifico L, Cantisani V, Ricci P, Osborn JF, Schiavo E, Anania C, Ferrara E, Dvisic G, Chiesa C (2008). Nonalcoholic fatty liver disease and carotid atherosclerosis in children. Pediatr Res.

[19] Dick TJ, Lesser IA, Leipsic JA, Mancini GB, Lear SA (2013). The effect of obesity on the association between liver fat and carotid atherosclerosis in a multi-ethnic cohort. Atherosclerosis.

[20] Akabame S, Hamaguchi M, Tomiyasu K, Tanaka M, Kobayashi-Takenaka Y, Nakano K, Oda Y, Yoshikawa T (2008). Evaluation of vulnerable coronary plaques and non-alcoholic fatty liver disease (NAFLD) by 64-detector multislice computed tomography (MSCT). Circ J.

[21] Loria P, Lonardo A, Targher G (2008). Is liver fat detrimental to vessels?: intersections in the pathogenesis of NAFLD and atherosclerosis. Clin Sci (Lond).

[22] Alisi A, Manco M, Vania A, Nobili V (2009). Pediatric nonalcoholic fatty liver disease in 2009. J Pediatr.

[23] Sert A, Pirgon O, Aypar E (2013). Relationship between aspartate aminotransferase-to-platelet ratio index and carotid intima-media thickness in obese adolescents with non-alcoholic fatty liver disease. J Clin Res Pediatr Endocrinol.

[24] Schwimmer JB, McGreal N, Deutsch R, Finegold MJ, Lavine JE (2005). Influence of gender, race, and ethnicity on suspected fatty liver in obese adolescents. Pediatrics.

[25] Fracanzani AL, Burdick L, Raselli S, Pedotti P, Grigore L, Santorelli G, Valenti L, Maraschi A, Catapano A, Fargion S (2008). Carotid artery intima-media thickness in nonalcoholic fatty liver disease. Am J Med.

[26] Cook S, Auinger P, Li C, Ford ES (2008). Metabolic syndrome rates in United States adolescents, from the National Health and Nutrition Examination Survey, 1999-2002. J Pediatr.

[27] Fallo F, Dalla Pozza A, Sonino N, Lupia M, Tona F, Federspil G, Ermani M, Catena C, Soardo G, Piazza L, Bernardi S, Bertolotto M, Pinamonti B, Fabris B, Sechi LA (2009). Non-alcoholic fatty liver disease is associated with left ventricular diastolic dysfunction in essential hypertension. Nutr Metab Cardiovasc Dis.

[28] Hurjui DM, Nita O, Graur LI, Mihalache L, Popescu DS, Hutanaşu IC, Ungureanu D, Graur M (2012). Non-alcoholic fatty liver disease is associated with cardiovascular risk factors of metabolic syndrome. Rev Med Chir Soc Med Nat Iasi.

[29] Alam S, Noor-E-Alam SM, Chowdhury ZR, Alam M, Kabir J (2013). Nonalcoholic steatohepatitis in nonalcoholic fatty liver disease patients of Bangladesh. World J Hepatol.

[30] Peterson LR, Herrero P, Schechtman KB, Racette SB, Waggoner AD, Kisrieva-Ware Z, Dence C, Klein S, Marsala J, Meyer T, Gropler RJ (2004). Effect of obesity and insulin resistance on myocardial substrate metabolism and efficiency in young women. Circulation.

[31] Atabek ME (2013). Possible misinterpretations of nonalcoholic fatty liver disease and intra-abdominal fat in prepubertal children born small for gestational age. Horm Res Paediatr.

